# Data Analyses and Modelling for Risk Based Monitoring of Mycotoxins in Animal Feed

**DOI:** 10.3390/toxins10020054

**Published:** 2018-01-26

**Authors:** H.J. (Ine) van der Fels-Klerx, Paulien Adamse, Ans Punt, Esther D. van Asselt

**Affiliations:** RIKILT Wageningen Research, Akkermaalsbos 2, 6708 WB Wageningen, The Netherlands; paulien.adamse@wur.nl (P.A.); ans.punt@wur.nl (A.P.); Esther.vanasselt@wur.nl (E.D.v.A.)

**Keywords:** contaminant, aflatoxin B_1_, deoxynivalenol, feed, trend analyses, risk, model

## Abstract

Following legislation, European Member States should have multi-annual control programs for contaminants, such as for mycotoxins, in feed and food. These programs need to be risk based implying the checks are regular and proportional to the estimated risk for animal and human health. This study aimed to prioritize feed products in the Netherlands for deoxynivalenol and aflatoxin B_1_ monitoring. Historical mycotoxin monitoring results from the period 2007–2016 were combined with data from other sources. Based on occurrence, groundnuts had high priority for aflatoxin B_1_ monitoring; some feed materials (maize and maize products and several oil seed products) and complete/complementary feed excluding dairy cattle and young animals had medium priority; and all other animal feeds and feed materials had low priority. For deoxynivalenol, maize by-products had a high priority, complete and complementary feed for pigs had a medium priority and all other feed and feed materials a low priority. Also including health consequence estimations showed that feed materials that ranked highest for aflatoxin B_1_ included sunflower seed and palmkernel expeller/extracts and maize. For deoxynivalenol, maize products were ranked highest, followed by various small grain cereals (products); all other feed materials were of lower concern. Results of this study have proven to be useful in setting up the annual risk based control program for mycotoxins in animal feed and feed materials.

## 1. Introduction

According to Regulation (EC) No 882/2004, Member States in Europe should establish and implement multi-annual control programs for contaminants in feed and food materials and derived products, to ensure that checks are regular and proportional to the risk for animal and human health [1]. In the Netherlands, descriptive (including trend) analyses of historical monitoring results and risk modelling are performed with the aim to obtain insights into which combinations of contaminant-feed materials are of highest concern. Data analyses focuses on occurrence of contaminants, using historical monitoring data such as, for example, on heavy metals in feed products [2]. Risk modelling focuses on estimating the consequences on animal and human health due to the presence of the contaminant in feed and feed materials [3]. The combined results are used by the National Food and Consumer Product Safety Authority (NVWA) for setting priorities for the multi-annual control program for contaminants in feed and feed materials each year.

This study aimed to prioritize feed and feed materials for risk based monitoring of deoxynivalenol and aflatoxin B_1_ in the Netherlands, by descriptive statistical analyses and risk modelling using historical monitoring results combined with other data sources.

## 2. Results and Discussion

### 2.1. Data: Overview of Numbers of Samples

The total dataset obtained for the aims of this study included 35,119 sample results over the study period 2007–2016; these included 11,834 records for deoxynivalenol and 23,285 records for aflatoxin B_1_. The origin of the records over public and private was about 1:3. Table 1 presents the number of samples per year for aflatoxin B_1_ and deoxynivalenol.

### 2.2. Descriptive Data Analyses

Results of the descriptive data analyses, including trend analyses over time, of concentrations of aflatoxin B_1_ and deoxynivalenol in animal feed and feed materials were examined for each feed product and toxin, separately. Next, the results were summarized for the presence of aflatoxin B_1_ and deoxynivalenol in feed and feed materials, using four different metrics and classified for their priority (low, medium, high), as described in the Material and Methods section.

### 2.3. Aflatoxin B_1_

**Figure 1:**
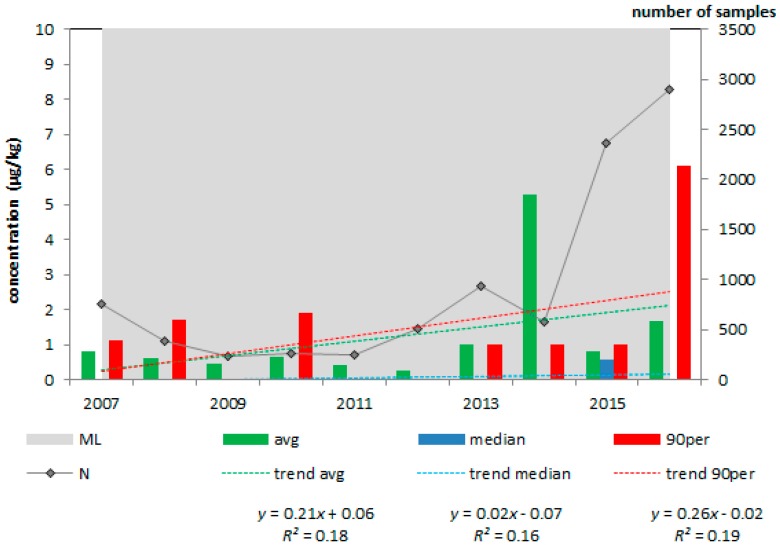
Average, median and 90th percentile concentration of aflatoxin B_1_ in maize and maize products in the period 2007–2016, N = 9160; ML = 20 µg/kg.

**Figure 2:**
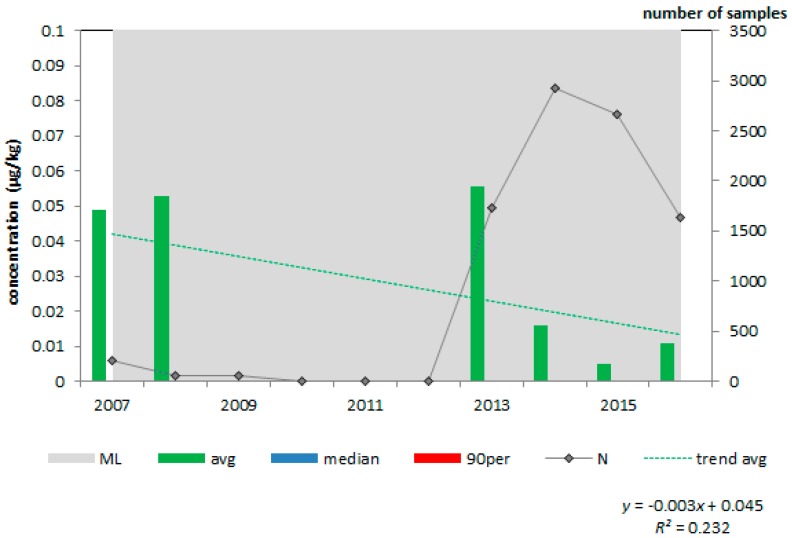
Average, median and 90th percentile concentration of aflatoxin B_1_ in compound feed for dairy cattle in the period 2007–2016, N = 9268; ML = 5 µg/kg.

For each animal feed and feed material, the average, median and 90th percentile concentration of aflatoxin B_1_ in the period 2007–2016 were plotted. For illustration purposes, Figure 1 and Figure 2 present the results for maize and maize products (Figure 1) and compound feed for dairy cattle (Figure 2).

As can be seen from Table 1 and the two figures, the number of samples collected from animal feed and feed material in the last years (starting in 2013) was much higher than in the earlier years of the period considered. In fact, in the years 2008–2012, only a few samples were collected from compound feed for dairy cattle. The increase in the number of samples is the result of a new strategy of the feed industry, after the aflatoxin incident in March 2013 in the Netherlands, as described by De Rijk et al. [4], to collect more maize samples for aflatoxin B_1_ analyses. In none of the samples, the average, median or 90th percentile concentration of aflatoxin B_1_ was higher than the EC Maximum Limit (ML) for the respective animal feed or feed material [5]. The 90th percentile aflatoxin B_1_ concentration in maize and maize products was much higher in 2016 than in the earlier year. In this year, 300 samples had a concentration of 6 or higher, of which six samples had a concentration above the ML. The maize and maize product samples originated from South and North America and East Europe.

The average aflatoxin B_1_ concentration decreased significantly during the study period in sorghum, sunflower seed expeller/extracted, coconut/copra expeller/extracted, maize gluten feed, soya hulls, rumen-protected soya extracted, soya bean and manioc. In the remaining animal feeds and feed materials, no significant increase or decrease of the average aflatoxin B_1_ concentration during the study period was observed.

After evaluation of the four summarizing metrics (Table 2), nearly all animal feeds and feed materials were ranked as low priority (III), except for groundnuts which had a high (I) priority and for both feed materials (especially maize and maize products and several oil seed products) and complete/complementary feed excluding dairy cattle and young animals which were classified as medium (II) priority. For groundnuts 265 RASFF notifications were recorded. In addition, high concentrations of aflatoxin B_1_ were found in this feed material, with 20% of the samples exceeding the ML and, therefore, this feed material was classified as high priority. Groundnuts are often used for bird feed. Several oil seed products, including products of sunflower seeds, palmkernel, soya bean and coconuts, were classified medium priority given the relative high number of RASFF notifications in the period 2007–2016 and the aflatoxin B_1_ concentration in the Dutch monitoring results (Table 2). Both maize and maize products and other feed materials were classified medium priority due to the high number of RASFF notifications, particularly for the former group of products (57 RASFF notifications, see Table 2). Note that the trend in aflatoxin B_1_ concentration in the remaining group of feed materials, thus excluding the ones mentioned separately in Table 2, over time decreased. RASFF notifications, causing the medium priority, originate from any EU country. Apparently, aflatoxin B_1_ contamination of this group of feed materials used in feed production in the Netherlands is under control; the medium priority implies that the contamination of this ingredient should have particular management attention.

Complete and complementary feed excluding dairy cattle and young animals also was classified as high priority because of RASFF notifications. Five of the six RASFF notifications in this animal feed related to bird feed. The fifth notification concerned a compound feed not further specified in RASFF. In the current dataset of historical monitoring results, only 16 sample results referred to groundnut (or derived products); 11 of these had a reported aflatoxin B_1_ concentration below the LOQ. The five sample results with an aflatoxin B_1_ concentration above the LOQ were all from 2008; four of these had a concentration (24, 42, 67 and 905 µg/kg) above the ML of 20 µg/kg. Other complete and complementary feeds were classified as low priority.

### 2.4. Evaluation of Deoxynivalenol Occurrence

**Figure 3:**
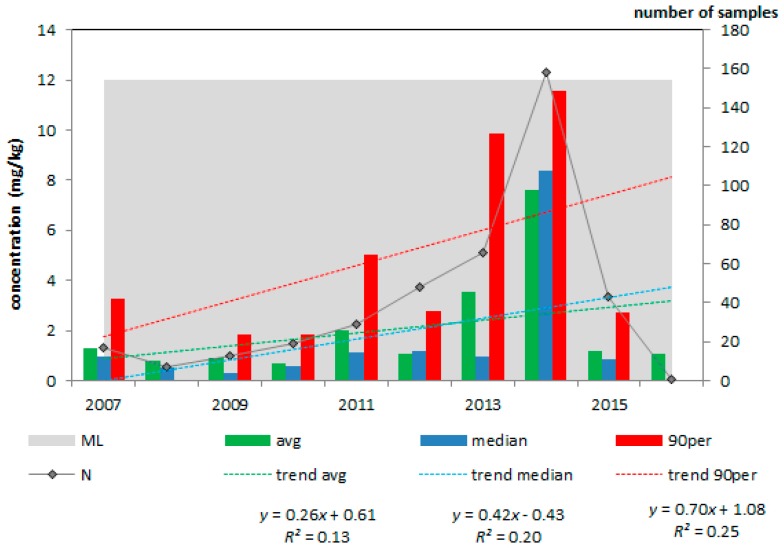
Average, median and 90th percentile concentration of deoxynivalenol in maize by-products; 2007–2016, N = 401; EC guidance value = 12 mg/kg.

**Figure 4:**
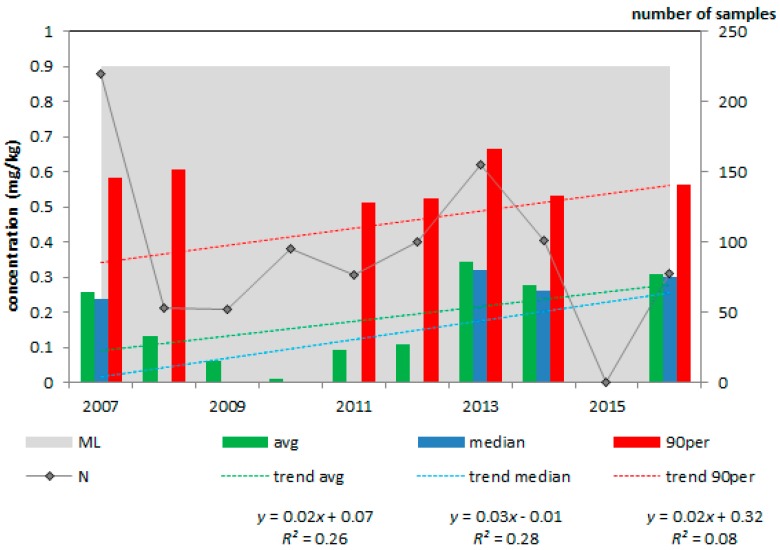
Average, median and 90th percentile concentration of deoxynivalenol in complementary and complete feedingstuffs for pigs; 2007–2016, N = 931; EC guidance value = 0.9 mg/kg.

**Figure 5:**
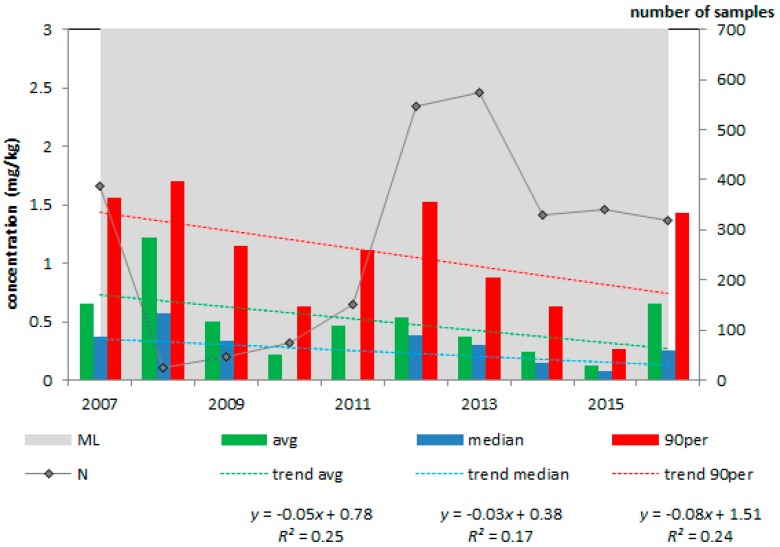
Average, median and 90th percentile concentration of deoxynivalenol in wheat and wheat products; 2007–2016, N = 2790; EC guidance value = 8 mg/kg.

The concentration of deoxynivalenol in animal feeds and feed materials varied between the study years, as can be seen from Figure 3, Figure 4 and Figure 5 (included here as example).

A high deoxynivalenol concentration was present in 2014 in maize by-products (Figure 3) but this concentration dropped to lower levels in 2015. Though averages, medians and 90th percentile values did not exceed the respective EC guidance values [6], individual samples did, as can be seen from Table 3.

After evaluation of the four metrics (Table 3), maize by-products were classified as high (I) priority. The average deoxynivalenol concentration was 34% of the EC guidance value (of 12 mg/kg) and in 3.2% of the more than 400 samples the deoxynivalenol concentration exceeded this guidance value. In none of the feed and feed materials (including maize by-products, Figure 3, Table 3), the deoxynivalenol concentration increased or decreased significantly during the period 2007 to 2016. Complementary and complete feedingstuffs for pigs (Figure 4, Table 3) were classified as medium (II) priority because one metric was above its threshold, i.e., the average deoxynivalenol concentration was 23% of the EC guidance value (0.9 mg/kg). The other products were ranked as low priority (III).

### 2.5. Risk Model

**Figure 6:**
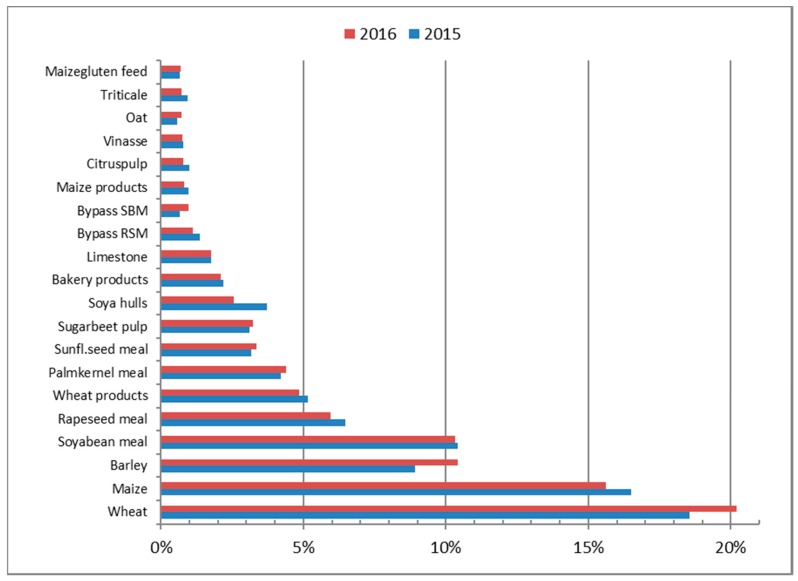
Relative use of feed ingredients for compound feed production in the Netherlands, in 2015 and 2016.

The total volume of feeds used in the Netherlands in 2016 was 16.5 Mt, which was about the same as in 2015. However, slight differences in volumes of some individual feed materials were observed (Figure 6): in 2016, less corn was used and more wheat and barley, as compared to 2015.

### 2.6. Consequence Factors

Values of the consequence factor for aflatoxin B_1_ on animal health were 0.01 for all 14 animal species. For human health, the values were 1 for dairy cows and for sheep and goats (for consumption of milk) and 0.001 for the remaining 11 animal species. The value of 0.001 was used because there is no ML established for food products derived from these latter animal groups.

For deoxynivalenol, consequence factors for animal health were 1 for all four pig species (piglets, growing pigs, gilts and sows) and 0.1 for the remaining 10 animal species. Deoxynivalenol consequence for human health factors were 0.001 for all animal species. Because there are no MLs established for deoxynivalenol in products from animal origin they were presumed not to be a problem and hence given this lowest value.

### 2.7. Prevalence Factors

Aflatoxin country factors were high for all regions, except for Central and North-West Europe and Oceania, which had a low value. For deoxynivalenol, country factors were high for Africa, North America, North Asia, Central and North-West Europe, whereas for South-America, South-East Asia, Mid-East, East and Southern Europe, these values were low.

All feed materials that originated from countries with a low country value were assigned the lowest prevalence value of 0.01. Further evaluation of feed materials that were sourced from countries with a high country value showed that, for aflatoxins, the prevalence value was high (value 1) for maize products, rice, sunflower and products thereof, groundnut and cottonseed expeller. All other cereals (including maize), coconut, palm and products thereof had a prevalence value of 0.1 and all remaining feed materials were given the value 0.001. For deoxynivalenol, maize products had a prevalence value of 1, all other cereals and cereal products (except maize products) had the value 0.1 and all remaining feed materials the value 0.01.

### 2.8. Feed Materials Ranking for Aflatoxin B_1_

**Figure 7:**
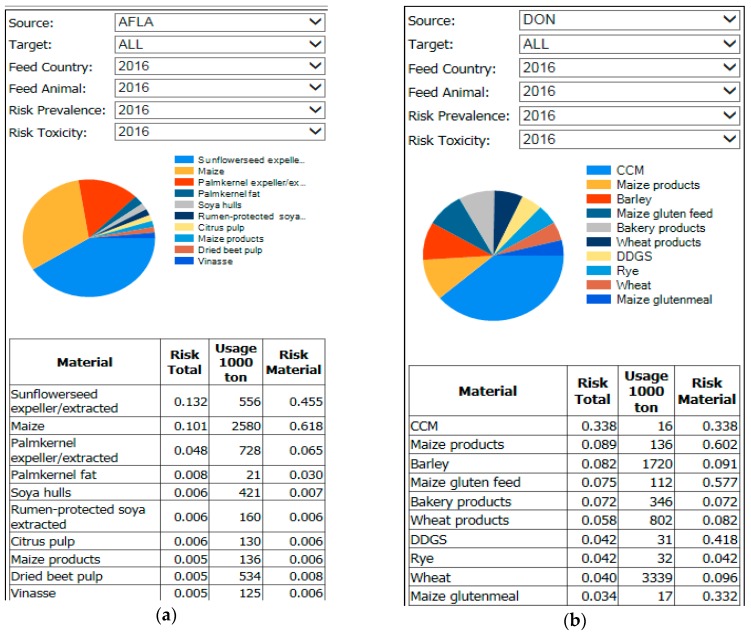
Results (top 10) for ranking feed materials for aflatoxin B_1_ (**a**) and deoxynivalenol (**b**), based on the RiskFeed model using feed material data for 2016.

Results of the RiskFeed model for aflatoxin B_1_ include the ranking of feed materials for monitoring priority based on the consequence of the presence of this toxin in feed materials on animal and human health, in combination with its prevalence in the feed materials (Figure 7). Results showed that sunflower expeller/extracted, maize and palmkernel products were ranked highest. For maize, this is mainly due to the high volume used for compound feed production in combination with its high prevalence (value 1). The high ranking of sunflower expeller/extracted can be explained by its high prevalence factor (value 1) in combination with the use of this feed materials for the production of compound feed for dairy cattle, which had a consequence factor of 0.01 for animal and 1 for human.

### 2.9. Feed Materials Ranking for Deoxynivalenol

The priority ranking of feed materials for deoxynivalenol, as based on the RiskFeed model (Figure 7), showed that the top 10 ingredients with the highest ranking all were cereal grains or related products. This is mainly due to the prevalence factors of these ingredients together with their high volumes used in animal feed production. Maize (including corn cob mix), barley and wheat (products) were ranked highest, which is due to the high prevalence factor value for maize products (value of 1) and the medium prevalence factor value for the other cereals and derived products combined with the high consumption of these feed materials by pigs which are sensitive to this mycotoxin (high animal health consequence value). For instance, the medium prevalence value for wheat was 0.1, as based on evaluation of the occurrence in the period 2012–2016 (presented in Figure 5). All remaining feed materials were not considered a problem in this period and had a prevalence value for deoxynivalenol of 0.01.

In this study, national monitoring data and RASFF notifications were used to observe the occurrence of aflatoxin B_1_ and deoxynivalenol in both feed materials and animal feeds. The monitoring data included the combined results from mycotoxin analyses done by both the government and feed companies. From the three steps of sample collection, sample preparation and sample analyses for mycotoxins, in particular for aflatoxins (which are heterogeneously distributed) variability in the sample collection step is contributing most to the total variability, followed by the sample analyses step [6]. For both sample collection and sample analyses, approved guidelines have been followed and accredited analytical methods used, by both the government and industry. However, there are slight differences between the procedures followed by government and industry. Also, some samples were collected on a risk-based approach. These factors may have introduced some bias in the data used.

Based on evaluating the results, a prioritization for monitoring was made. This does not necessarily mean these feed products are of a concern, rather they could be a concern and therefore should have inspection attention. Secondly feed ingredients were further prioritized not only considering mycotoxin occurrence but also risk for animal and human health by their use in animal feed production. Results of both descriptive analyses for feed materials and the RiskFeed model were quite comparable but some differences in the prioritization of ingredients were seen. These differences can be explained, amongst others, by the extent to which they are used in feed production for specific animal species, their volumes and countries of origin. For instance, groundnuts were ranked high priority, based on the descriptive analyses. However, the RiskFeed model did not result into a top-10 ranking of this feed material. The reason is that ground nuts are mainly used for bird feed and this type of feed has no effect on human health (was not considered in the RiskFeed model).

Results of this study, both from the descriptive data analyses and the RiskFeed model, were presented and discussed with the National Food and Consumer Product Authority (NVWA) in the Netherlands, in November 2017. These results were considered an important source of information for setting up the annual risk based control program for mycotoxins in feed and feed materials for 2018. The next step is to determine the number of samples that should be collected from each ingredient. This step is not part of the RiskFeed model but is currently done by the competent authority and feed companies themselves. Future research could focus on adding sample size calculations as a next step to the RiskModel.

The model has been made flexible and user-friendly so that input data can be updated or changed easily. This is considered useful for scenario (what-if) analyses, annual updating and/or using dedicated datasets.

## 3. Conclusions

Results of this study relate to prioritizing animal feeds and feed materials for deoxynivalenol and aflatoxin B_1_ monitoring on the basis of (1) the occurrence of the two toxins in these feed products using descriptive statistical analyses of historical monitoring results and RASFF notifications; and (2) estimating consequences for animal and human health due to the presence of mycotoxins in feed materials used for feed production. Based on evaluation of occurrence data and RASFF notifications, groundnuts had a high priority for aflatoxin B_1_ monitoring. Other feed materials, particularly maize and maize products and several oil seed products and complete/complementary feed excluding dairy cattle and young animals were considered medium priority for aflatoxin B_1_ monitoring. When also considering health consequences, feed materials that were of highest priority included sunflower seed expeller/extracts, palmkernel expeller/extracts and maize. For monitoring deoxynivalenol, maize by-products were considered high priority and complete and complementary feed for pigs were of medium priority, using the occurrence and RASFF data. Also considering health effects showed that feed materials of highest concern were maize products, small grain cereals and products thereof. These results have shown to be useful as one of the information source for underpinning the risk based control plan for mycotoxins in feed in the Netherlands.

## 4. Material and Methods

Statistical (descriptive) analyses of historical monitoring results was performed to obtain insights into the occurrence of aflatoxin B_1_ and deoxynivalenol in feed and feed materials. Based on the results, the feed (materials) are classified as low, medium or high priority for monitoring. In order to obtain insights into monitoring priority of the feed materials also considering consequences of the presence of the toxin in feed and feed materials on animal and human health, a risk model (named RiskFeed model) was used. This model integrates occurrence data with other data sources to provide a ranking of feed materials as output. Data used, descriptive data analyses and risk modelling are outlined below.

### 4.1. Monitoring and Other Data

For both the descriptive data analyses and the risk modelling, government data as well as data from feed industries and open data were used. Government data included the results of the multi-annual monitoring program from the period 2007–2016. These data referred to the sample results from the official control of the two mycotoxins feed and feed materials. The methods for collection of samples, the preparation of samples and sample analyses followed the requirements that were prescribed by the European Commission, as laid down in Commission Regulation (EC) No 152/2009 [7] and the general requirement laid down in Regulation (EC) No 882/2004 [1] and Regulation (EU) 2017/625 [8]. In practice LC-MS/MS, accredit method was used for sample analyses. Through a cooperation with SecureFeed—a Dutch organization in which approximately 400 companies that supply animal feed (feed materials, compound feed, forage and roughage, moist feed materials, mineral feed etc.) directly to livestock farmers in the Netherlands participate to invest in the assurance of safe feed—the provision of private data for the aims of the current study was ensured. Data provided included the results from monitoring mycotoxins in feed and feed materials by private companies (SecureFeed members) in the period 2007–2016. Sample collection through the SecureFeed program followed the GMP+ procedures, as laid down in GMP BA 13 “Minimal requirements for sampling”. Samples were analysed in SecureFeed approved laboratories, using accredited methods (by the Dutch Accreditation Council, see www.rva.nl). In practice, HPLC with fluorescence or LS-MS/MS were used. Furthermore, data from the Rapid Alert System for Feed and Food (RASFF) related to the occurrence of mycotoxins in feed and feed materials in the same period were extracted from the RASFF portal (https://webgate.ec.europa.eu/rasff-window/portal).

### 4.2. Descriptive Analyses

#### 4.2.1. Statistical Data Analysis

Product names were harmonized to enable combining data from the different sources into one dataset. The products were grouped according to the groups used for defining the EC maximum limit (ML) for aflatoxin B_1_ in animal feed and feed materials [2] according to Directive 2002/32/EC [5] (last amended by Regulation (EU) 2015/186) or the EC guidance values for deoxynivalenol in animal feed and feed materials according to Recommendation 2006/576/EC (EC, 2006) [9]. This enabled grouping products with the same ML or guidance value. Where applicable, subgroups were used to highlight specific feed and feed materials that differed from the rest of the group.

Because the data originated from multiple years and suppliers the limits of quantification (LOQ) of the analytical method used varied. However, in general the LOQs for aflatoxin B_1_ and deoxynivalenol were 1 µg/kg and 0.1 mg/kg, respectively. Values reported as to be below the LOQ were replaced by zero for the statistical analysis. Apparent outliers, i.e., samples with an exceptionally high concentration, were examined individually; they were excluded from the dataset when they appeared to be the result of targeted (i.e., follow up) sampling [10].

For aflatoxin B_1_ and deoxynivalenol, four different statistical metrics were calculated using descriptive analysis of the monitoring results. These included: the average (avg), median (P50), 90th (P90) percentile and the percentage of samples exceeding the ML (% > ML), per feed or feed material group or sub-group. The Pearson Correlation Coefficient (MS Excel^®^) was used to evaluate the significance of potential trends in the presence of the mycotoxin in the feed product over the time period considered. This was done for each of the average, median and 90th percentile of the presence of the mycotoxin. Trends with *R*^2^ values exceeding 0.30 were considered significant. In some trend analyses, 20 observations per year were considered minimum [11] but in the current analysis every amount of measurements per year is taken into account. However, sometimes the number of samples was too low to consider any trend to be significant.

Results of the statistical analyses and the RASFF notifications were studied in detail, per combination of mycotoxin—feed/feed material product group or sub-group, for each of the four criteria. Results were evaluated and summarized in separate tables per mycotoxin, with colours indicating the level of priority the feed or feed material should have in the national monitoring plan, based on the presence of the toxin in that feed material. The priority was considered high (I) when 10% or more of the samples exceeded the ML. The priority was also considered high (I) when two or more of the following criteria were fulfilled: average concentration higher than 20% of the ML (or recommendation in case of deoxynivalenol), more than 3% of the samples exceeding the ML, a significant increase of the average concentration between 2012 and 2016, and/or more than five RASFF notifications. The priority was considered medium (II) when only one of the criteria was fulfilled. With low priority (III) none of the criteria were fulfilled.

#### 4.2.2. RiskFeed Model

The RiskFeed model aims to rank, per mycotoxin, the various feed materials used for compound feed production, based on the potential consequence of the mycotoxin on animal and/or human health due to the presence of the particular mycotoxin in the ingredient considered, combined with the occurrence of the toxin in that feed material. The basic model is detailed in Van der Fels-Klerx et al. [3]; a summary is given below together with some recent model additions.

The RiskFeed model estimates the potential consequence of the presence of the mycotoxin in compound feed on animal and human health. To this end, the presence of the mycotoxin in compound feed for 14 different animal species is calculated, based on the presence of the toxin in the feed materials used in the specific compound feed and inclusion rates of feed materials in each of the compound feeds. Volumes of feed materials used, countries of origins and prevalence of the mycotoxin in the ingredient in the country of origin is accounted for. The 14 animal species considered include: piglets, growing-finishing pigs, rearing gilts, sows, broiler chickens, laying hens, broiler breeders, dairy cows, dairy calves-heifers, veal calves, veal cattle, sheep, goats and horses.

As input the RiskFeed model uses: yearly volumes of all feed materials used for the production of compound feeds (a); per country of origin (b); used portions of feed materials for compound feed production per animal type (c); presence of the toxin in each feed material (prevalence factor d); and the consequence of the presence of the mycotoxin in the compound feed on animal and human health (consequence factor e). The volume of each compound feed material (in kton) is considered a continuous variable from which the ^10^log value is taken. The other model factors (b–d) can have values between 0–1. Factors b and c can be assigned any value between 0–1, whereas factors d and e can be assigned one of four values of 0.001, 0.01, 0.1 and 1. The model then multiplies all factors with each other to arrive at the overall risk value per feed material; this risk value can be used to rank the feed materials for priority in the multi-annual control program. In contrast to the descriptive data analyses, the RiskFeed model only ranks feed materials (not animal feeds). Relative to the model presented in Van der Fels-Klerx et al. [3] the procedure to estimate the prevalence factor and the consequence factor for the two mycotoxins aflatoxin B_1_ and deoxynivalenol was modified. The modifications are presented below.

### 4.3. Prevalence Factor

As the fungi that produce aflatoxin B_1_ and deoxynivalenol do not occur in all countries, the likelihood for the presence of aflatoxin B_1_ and deoxynivalenol in feed materials was first assessed per country/region and second per feed material, using data and information from the last five years. The region likelihood for each of aflatoxin B_1_ and deoxynivalenol was assessed using literature data [12,13], legislation specifying countries for monitoring on aflatoxins, including Regulations (EC) No. 669/2009 [14] and (EC) No. 884/2014 [15] and expert opinion. Regions could be assessed as low or high likelihood for each of aflatoxin B_1_ and deoxynivalenol.

All ingredients sourced from countries with a low likelihood for the toxin were assigned a 0.01 value for the prevalence factor. Ingredients from countries that had a high likelihood for the occurrence of the toxin, were examined in more detail to arrive at the prevalence factor value. For those ingredients, prevalence factors values were based on total scores for five different criteria of which four were based on monitoring data, analogous to Adamse et al. (2017) and one was based on literature and expert information. A subset of the monitoring data of descriptive analyses was used related to the years 2012–2016, as well as RASFF data from the same years. Feed materials were assigned a score 1 for each of the following four criteria: (1) more than 3% of the samples exceeded the ML; (2) the average concentration was more than 20% of the ML; (3) a positive trend (*R*^2^ ≥ 0.3) and; (4) more than four RASFF notifications. For the 5th criteria, literature and experts were consulted. If these information sources indicated that there was evidence of high concentrations of mycotoxins in the ingredient, a score of 2 was assigned, if there were no indications of mycotoxin presence in the ingredient, a score of 0 was obtained and a score of 1 was obtained if there were some indications that mycotoxins can be present in the ingredient. All scores were added: each ingredient could obtain a maximum of 6 points and a minimum of 0 points for the presence of the toxin. In case the total score was 3 or higher, the prevalence factor of the ingredient was assigned the value 1; in case the total score was 1 or 2, the prevalence factor was assigned the value 0.1; in case the total was zero, the prevalence factor was assigned the value 0.01.

#### 4.3.1. Consequence Factor

The consequence factor (d) is distinguished into a factor for animal and for human health. The consequence for human health incorporates the presence of the mycotoxin in animal derived food products (e.g., milk, eggs) and the potential health concern for humans after consumption of the particular animal derived food product. The consequence factor for animal health indicates the health concern of the mycotoxin for the particular animal type. Differences between animal types are allowed for. For example, pigs are more sensitive to deoxynivalenol as compared to dairy cows, so the consequence factor for animal health will be higher for pigs than for dairy cows. The consequence factor for aflatoxin B_1_ on human health, on the other hand, will be higher for dairy cows as compared to pigs, since aflatoxin B_1_ is transferred to dairy cows’ milk and is toxic to human health upon consumption of this milk.

Within the original RiskFeed model [3], the values of the consequence factors were based on expert judgment, considering available data and scientific literature on residue transfer and accumulation and toxic effects of the contaminant on animal and human. To make the estimation of the consequence factor more transparent and objective, rather than using expert judgement only, a data driven method was established. To this end, the impact of each contaminant on animal or human health is estimated based on the ratio between exposure via feed/food intake and the dose levels that induces adverse health effects in animals or humans. Based on this ratio, the consequence factor is assigned a value 0.01, 0.1 or 1. The consequence factor is (only) assigned the value 0.001 in case a ML or guidance limit is not established for the particular mycotoxin. Comparable to general toxicological safety evaluations, exposure from food or feed that are close to the dose levels that induce adverse health effects (i.e., low ratio between adverse effect levels and exposure levels) were considered of high concern. In that case, the consequence factor was assigned the value 1. In a similar way, a median (consequence factor of 0.1) and low (consequence factor of 0.01) concern for health risk were defined. Both the derivation of information on the general exposure levels of animal and human to each of aflatoxin B_1_ and deoxynivalenol and the safe dose levels were based on evaluations performed by EFSA [16,17,18] and JECFA [19]. These include the reported no observed adverse effect levels (NOAELs) for animal health and the TDI (tolerable daily intake) or VSD (virtual safe dose) for human health.

Some assumption had to make to fill in data gaps. For example, the dose levels that are linked to adverse health effects are not always available for all animal species considered and—in that case —were based on related species (e.g., extrapolation of cattle data to goats or sheep) or set at the lowest available level within the range of species. For animal exposure, EFSA reported median concentrations in feed were considered, when available, which were then compared with the dose levels that induce adverse health effects in the animals (expressed a ng/kg feed). A ratio between TDI and exposure higher than 75 was considered to be of low concern (consequence factor 0.01); 25–75 of median concern (consequence factor 0.1); and <25 of high concern (consequence factor 1). For humans, EFSA reported median concentrations in animal derived products (i.e., milk, eggs, or meat) were considered. These were in turn converted to a human exposure estimate (mg/kg bw/day), based on (i) a worst-case estimation of the consumption of these products (derived from the EFSA consumption database) and (ii) a bodyweight of 70 kg. An estimated human intake that is close to the TDI or VSD (i.e., within 2-fold) was considered to be of high concern (consequence factor 1), a ratio between 2 and 5 of median concern (consequence factor 0.1) and a ratio higher than 5 of low concern (consequence factor 0.01).

#### 4.3.2. Ingredient Data

Data on the volumes of ingredients used in the year 2016 were obtained from SecureFeed. These data concern all feed materials used for compound feed production (over 95% of national production) in the Netherlands. Eurostat data of the year 2016 were used to obtain the information (ratios) on countries of origins for each of the feed materials. Portions of ingredients used in the production of compound feeds for the various animal types were obtained using the linear optimization module for compound feed production [3], using market prices of feed ingredients in 2016. Based on the excel version of the RiskFeed model, developed earlier, a user-friendly PC-based application of the model was constructed in the course of this study.

#### 4.3.3. Calculations

All input data were entered into the online model. Model output include the rankings of the feed ingredients with the highest expected consequences for animal health, human health and their combination for each of aflatoxin B_1_ and deoxynivalenol.

## Figures and Tables

**Table 1:** Overview of numbers of monitoring results for deoxynivalenol (DON) and aflatoxin B_1_ (AFB1) per year, public and private data, in the period 2007–2016.

Year	Public	Private	Public	Private
	DON	DON	AFB1	AFB1
2007	351	1429	236	1279
2008	321	76	223	531
2009	304	100	407	411
2010	423	151	454	417
2011	551	153	569	314
2012	517	1255	517	626
2013	504	1630	450	2675
2014	418	1415	393	3519
2015	303	914	294	5077
2016	411	608	375	4518
total	4103	7731	3918	19,367

**Table 2:** Summary of evaluation of occurrence of aflatoxin B_1_ in animal feed and feed materials between 2007 and 2016.

Products Intended for Animal Feed	ML * (µg/kg)	N (2007–2016)	Average (% of ML *)	% > ML *	Trend Average (*R*^2^)	RASFF (2007–2016)	Priority
Feed materials with the exception of:	0.02	2562	0.8	0.1	neg _(0.39)_	**6**	II
—maize and maize products	0.02	9160	7	0.3	0 _(0.18)_	**57**	II
—groundnuts	0.02	20	**261**	**20**	0 _(0.16)_	**265**	I
—sunflower seed-, palmkernel-, soya bean- and coconut products	0.02	1333	**1**	**0.1**	0 _(0.19)_	**29**	II
Complete and complementary feed with the exception of:	0.01	14	0	0	0 _(0.00)_	**6**	II
—compound feed for cattle (except dairy cattle and calves), sheep (except dairy sheep and lambs), goats (except dairy goats and kids), pigs (except piglets) and poultry (except young animals)	0.02	794	0.3	0	0 _(0.00)_	0	III
—compound feed for dairy cattle and calves, dairy sheep and lambs, dairy goats and kids, piglets and young poultry animals	0.005	9406	0.4	0	0 _(0.10)_	1	III
Other (premix, mineral mix, amino acid, mono calcium phosphate, water)	no ML	46	-	-	0 _(0.10)_	0	III

* Maximum level (ML) as presented in [5].

**Table 3:** Summary of evaluation of occurrence of deoxynivalenol in animal feed and feed materials between 2007 and 2016.

Products Intended for Animal Feed	GV *** (mg/kg)	N (2007–2016)	Average (% of GV ***)	% > GV ***	Trend Average (R^2^)	RASFF (2007–2016)	Priority
Feed materials (excl. cereals and cereal products	no GV	1982	-	-	0 _(0.19)_	0	III
Cereals and cereal products * with the exception of maize by-products	8	8357	7.2	0.3	0 _(0.02)_	0	III
Maize by-products	12	406	**34**	**3.2**	0 _(0.13)_	0	I
Complete and complementary feed with the exception of:	5	154	5.2	0	0 _(0.12)_	0	III
*subset: poultry feed*	5	102	2.7	0	0 _(0.19)_	0	III
—complementary and complete feedingstuffs for pigs	0.9	931	**23**	1.6	0 _(0.26)_	0	II
—complementary and complete feedingstuffs for calves (<4 months), lambs and kids **	2	0	0	0	n/a	0	n/a
Other (premix, mineral mix, amino acid, animal fat)	no GV	4	-	-	0 _(0.00)_	0	III

* The term ‘Cereals and cereal products’ includes not only the feed materials listed under heading 1 ‘Cereal grains, their products and by-products’ of the non-exclusive list of main feed materials referred to in part B of the Annex to Council Directive 96/25/EC of 29 April 1996 on the circulation and use of feed materials (OJ L 125, 23.5.1996, p. 35) but also other feed materials derived from cereals in particular cereal forages and roughages. ** Only 1 sample of feed for calves in the dataset (with deoxynivalenol level < LOQ). It could not be determined whether the calves were <4 months. *** Guidance value (GV) as presented in [6].
